# Latent Class Analysis of Barriers to Care Among Emergency Department Patients

**DOI:** 10.5811/westjem.2018.11.40144

**Published:** 2019-02-04

**Authors:** Beau Abar, Ashley Holub, Steven Hong, Eric Aaserude, Vincent DeRienzo

**Affiliations:** University of Rochester Medical Center, Department of Emergency Medicine, Rochester, New York

## Abstract

**Introduction:**

Emergency department (ED) patients experience a variety of barriers to care that can lead to unnecessary or repeated visits. By identifying the patterns of barriers experienced by subsets of the ED patient population, future researchers might effectively design interventions to circumvent these barriers and improve care. This study sought to identify classes of individuals with regard to perceived barriers to care.

**Methods:**

Over a 10-week period, two medical students distributed surveys to eligible patients ≥18 years who presented to the ED. After consent, patients provided demographics data and rated their perceived access to care on nine specific items (scored 1–5). We used latent class analysis (LCA), a parametric clustering method, to determine patient groups. Demographic characteristics were then compared across classes.

**Results:**

We enrolled a total of 637 patients. Results of the LCA indicated that a six-class solution fit best: 1) low barriers (60%); 2) “work responsibility” barriers (13%); 3) economic-related barriers (10%); 4) “appointment difficulty” barriers (8%); 5) “illness and care responsibilities” barriers (6%); and 6) diverse barriers (2%). Patients in the low-barriers class were the oldest across classes (p<.001). Individuals in the low-barriers class were also more likely to be White (p=.015) and have private insurance (p<.001) than those in the “appointment difficulty,” “illness and care responsibilities,” and diverse barriers classes.

**Conclusion:**

LCA suggests there are six distinct classes of patients with regard to perceived access to care. These classes may be used as a potential starting point in designing targeted interventions for ED patients to improve continuity of care.

## INTRODUCTION

Overutilization of the emergency department (ED) has become a growing public health concern due to the burden placed on ED resources, space, and staff.^1^ There is a need for clarification of factors contributing to ED overuse in order for successful intervention design and implementation to offset this burden. Insurance status alone was once commonly believed to be the root cause of most of the ED burden; however, empirical evidence has consistently been unable to demonstrate this correlation. Other individual- and healthcare system-level barriers to care have been identified, leading to consistent use of the ED as a main point of care.^2^ Common barriers may include financial difficulties, logistical concerns associated with scheduling an appointment (e.g., transportation, work, or childcare responsibilities), and discomfort regarding interacting with providers.^2^,^3^ These barriers, however, do not exist in a vacuum, as they often cluster together within individual patients. Increasingly, studies have made use of latent class analysis (LCA), a parametric mixture modeling technique, to identify patterns of characteristics with which patients present as a way to better inform subsequent interventions.^4^–^7^ A subset of this literature has focused on patterns of perceived barriers to care.^8^–^10^

### Objective

Our goal was to build upon previous research by identifying classes of ED patients with differential patterns of perceived barriers to care. We then sought to examine differences across classes with regard to patient demographic characteristics. By identifying which patterns of perceived barriers are likely to occur within subsets of the ED population, a more-targeted intervention approach could be designed for the specific classes displaying elevated risk.

## METHODS

### Study Design, Setting, and Population

This survey study, conducted between June–August 2015, involved the screening of a convenience sample of adult patients (>18 years) presenting to the ED at Strong Memorial Hospital (Rochester, New York).

### Study Protocol

Two medical students were stationed in the ED for a total of ~70 hours per week for 10 weeks to recruit patients into the study. Representatives of the University of Rochester Medical Center (URMC)

The Emergency Department Research Associates (EDRA)^11^ program first approached all eligible patients >18 years of age, broadly introduced the study, and asked if patients would be willing to learn more about it. Exclusion criteria included (a) an inability to communicate in English, (b) presentation to the psychiatric ED, (c) presentations for intoxication, suicide attempt, mental health arrest or overdose, and (d) patients who had an Emergency Severity Index score of 1 and/or were in the critical care bay. The medical students would then present the study in detail to those who agreed, use a formalized procedure to determine capacity to consent, obtain written informed consent from eligible patients, and administer a brief survey including demographics and perceived access to care. Participant responses were recorded directly into a secure online data collection website.^12^ This study was approved by the Research Subjects Review Board at the URMC.

### Measurements

#### Demographic Characteristics

Age was reported in three bins: 1) 18–26; 2) 27–65; and 3) 65 years or older. Participants self-reported their gender (male, female, or other), race (White, Black/African American, Asian, Native American, Native Hawaiian/Pacific Islander, multiracial, or other), ethnicity (Hispanic or non-Hispanic), insurance status (no insurance, private insurance, Medicaid, Medicare, or other), and presenting complaint.

**Population Health Research Capsule**

What do we already know about this issue?

*Patients presenting to the emergency department (ED) often lack continuity of care due to observed and/or perceived barriers*.

What was the research question?

To what extent can we describe distinct subgroups of the ED patient population with differential patterns of barriers to care?

What was the major finding of the study?

*There are six distinct patterns of barriers emerged: 1) low barriers; 2) working barriers; 3) financial barriers; 4) appointment concerns; 5) illness concerns; and 6) many barriers*.

How does this improve population health?

*Understanding subsets of the ED patient population that experience distinct barriers to care can help facilitate more effective tailored interventions*.

#### Access to Care

Participants reported on the extent to which their ability to see a doctor in the prior year was limited by nine specific barriers.^13^ Items asked about the following: a) taking care of others (such as caring for a spouse or grandchildren); b) lack of insurance; c) difficulty finding transportation; d) doctor, clinic, or hospital bills; e) work responsibilities; f) fear that the doctor would discover a serious illness; g) feeling that the doctor is not responsive to the patient’s concerns; h) embarrassment about a potential illness; and i) confusion when trying to schedule an appointment. Response options were coded as 0 = “Not at All” and 1= “Very Little” to “A Whole Lot” (indicating some level of barrier). This measure has been previously used with ED populations.^14^ For the purpose of this study, any value greater than zero was coded as endorsement of the barrier.

### Data Analysis

We performed a series of LCAs using the set of barriers to care, with classes added until the best-fitting solution was identified. LCA is a clustering method whereby distinct unobserved subgroups (i.e., latent classes) of a sample/population are identified based on a series of responses to categorical items. The resulting groups are relatively homogeneous with regard to their patterns of response to the indicator items. Importantly, LCA improves upon older clustering methods by providing statistical justification for the class solution chosen. Specifically, the minimum Akaike Information Criteria (AIC)^15^ and Bayesian Information Criteria (BIC)^16^ values across solutions (e.g., two classes, three classes, etc.) are indicative of the statistically best-fitting class structure. Substantively, model selection was also guided by the distinguishability of profiles (e.g., extent of substantive overlap between classes) and the ability to interpret the resulting model.^17^ Once the best-fitting model was identified, we examined class differences in demographic characteristics using the auxiliary function in M*plus* v7.1, a latent variable modeling program (Muthén & Muthén, Los Angeles, California).^18^

## RESULTS

### Descriptive Analyses

The sample for this study was 636 consenting patients who completed a short survey. Patient characteristics are shown in Table 1. The gender distribution was fairly symmetrical, and age was normally distributed in this predominantly White sample (72%). More than half (55%) of the sample reported having private insurance. Overall, the most prevalent barriers reported in the sample were work responsibilities (23%), fear that a doctor would not be responsive to their concerns (20%), and transportation barriers (20%).

### Latent Class Analysis

When we conducted a binary LCA using M*plus*, we found discrepancy across the AIC and BIC with regard to the best-fitting model, as the AIC was lowest value in the six-class solution (5 class = 4518.60; 6 class = 4517.06; 7 class = 4517.12), while the BIC was lowest at the two-class solution (1 class = 4931.54; 2 class = 4659.02; 3 class = 4672.39). Given our goal of exploring potentially small subgroups of the ED population for whom unique patterns of barriers exist, we chose to follow the AIC and retain the six-class model. This choice was supported by the presence of highly distinguishable classes, as indicated by a model entropy value of 0.86. (Values greater than 0.80 indicate limited class overlap,)

The conditional probabilities of item endorsement for each of the six classes are presented in Figure, as are the overall probabilities of item endorsement. The majority of the sample (60%) fell into the low barriers class (class 1), citing minimal barriers to care. Class 2 (labeled the working barriers class, 13%) endorsed mainly issues concerning work responsibilities. Class 3 (the financial barriers class, 10%) was comprised of individuals who had primarily financial barriers, commonly endorsing items such as difficulty with hospital bills and lack of insurance. Class 4 (the appointment concerns class, 8%) was made up of individuals who had barriers pertaining to the actual appointment such as confusion scheduling the appointment, getting to the appointment, and feelings of doctor unresponsiveness at the appointment. Class 5 (the illness concerns class, 6%) was mainly concerned about other illnesses, as well as embarrassment over a potential illness. Class 6 (the many barriers class, 2%) represented a minority of participants, but it was the most extreme citing a large number of barriers prevalent in all class members including doctor, clinic or hospital bills, work responsibilities, fear of discovering another illness, and feelings that the doctor was not responsive to concerns. Embarrassment about potential illnesses was also highly reported (86%) among this class.

### Comparing Classes on Covariates

We then compared classes on a set of demographic and care-related covariates to provide greater context for the observed groupings (see Table 2). Results demonstrate that participants in the low barriers class were significantly older than individuals in any of the other classes (each pairwise comparison p<0.001). Individuals in the low barriers class were also more likely to be White and have private insurance than those in the appointment concerns, illness concerns, and many barriers classes. The highest rates of White or privately insured patients were seen in the working barriers class.

## DISCUSSION

We sought to identify mutually exclusive patterns of perceived barriers to care among ED patients. Results of a series of LCAs revealed six distinct classes, with the majority of the sample reporting little difficulty accessing care (60%). The smallest class identified (2%) was also the most extreme, strongly endorsing a very broad set of barriers to care. Similar results were observed in a study by Thorpe and colleagues 8 where three latent classes were used to describe patterns of barriers to care among older adults: 1) a majority class (75%) endorsing low barriers; 2) a class largely reporting logistical barriers (18%); and 3) a minority class (2%) with high likelihood of endorsing many barriers to care (including financial barriers similar to the current study). Other studies among behavioral health patients have presented a two-class solution, with a majority class with few barriers and a minority class with moderately high probabilities of many barriers.^9^,^10^

The current findings extend previous work in two important ways. First, we performed this study using a different population of patients (i.e., ED patients) who tend to have elevated difficulties accessing appropriate care, as evidenced by the smaller proportion of the sample reporting minimal barriers in the current study than in the referenced previous work. Second, the current study was better able than previous work to specify distinct groups of patients with differential patterns of barriers. Specifically, our study may be of particular value because the findings within a six-class solution provide much more granularity with regard to description and practical implications compared to previous studies. A reliance on splitting a sample into low, moderate, and high difficulty accessing care classes makes it hard to design meaningful interventions to address those at risk, as the interventions would likely be too general and inefficient to effect change. By identifying and describing five distinct patterns of elevated barriers, subsequent researchers may be able to design tailored interventions to mitigate the specific concerns presented by each class of patient.

These potential tailored/targeted intervention efforts are further strengthened by the results of the covariate analyses performed. Specifically, these analyses provided a great deal of additional description, with regard to patient demographics, to the latent classes observed. For example, racial minority patients were more common in the three classes reporting elevated barriers (appointment concerns, illness concerns, many barriers) than in the minimal barriers class, particularly over-representing the many barriers class. These same three barriers classes are over-representative of the subsets of individuals without private insurance. A more thorough depiction of the latent classes identified will further enhance subsequent intervention efforts seeking to improve continuity of care for ED patients.

## LIMITATIONS

There are several limitations to the current study that should be addressed. First, we relied on patient self-reports of perceptions of access to care, such that future research might seek to limit recall bias by using multiple methods of validating reports (e.g., confirmation using a separate screener; timeline follow-back of difficulties accessing care). Second, it is possible that this study may have underestimated the prevalence of several of the latent classes reporting barriers, particularly the many barriers class, as this is an ED sample in which those individuals with the most severe conditions, including severe psychiatric disturbances and substance use problems, may have been missed. These subsets of patients often experience significant difficulties accessing care. A related third concern is that the many barriers class was observed very infrequently, such that subsequent research is needed to better establish its validity. This concern is mitigated, however, by the previous work that has demonstrated a very similar class prevalence^8^ and the presented covariate analyses that distinguished this class from several other latent classes.

Fourth, a large majority of the sample was White and had insurance coverage, such that subsequent multisite work with more diverse population may be needed to confirm the class structure observed. Furthermore, the use of a convenience sample, rather than a fully consecutive sample, limits generalizability of findings to patients with lower-acuity complaints without altered mental status or psychiatric concerns (e.g., intoxicated, suicidal ideation). Patients with more acute and/or behavioral health presentations may demonstrate differential patterns of barriers to care that should be identified in follow-up research.

## CONCLUSION

The current study made use of advanced methods (ie, latent class analysis) to delineate six distinct patterns of barriers to care experienced by patients in the ED. By replicating these patterns in a more diverse sample of ED patients and effectively designing interventions to mitigate these specific patterns of barriers, researchers may be able to impact the continuity of care for the patients who present to the ED.

## Figures and Tables

**Figure:**
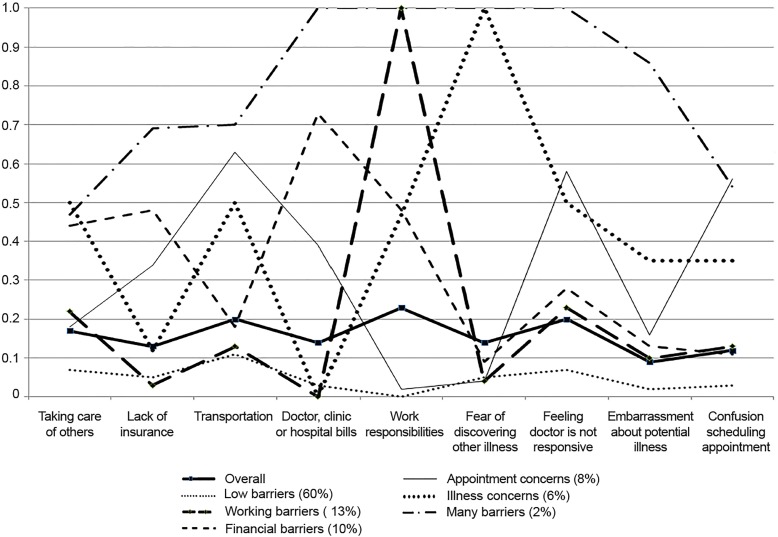
Conditional probabilities of endorsing barriers to care.

**Table 1:** Descriptive statistics of patients endorsing barriers to access of care.

	Frequency	%
Participant sex*		
Male	279	48%
Female	305	52%
Age		
18–26	122	19%
27–65	393	62%
65+	121	19%
Race		
White	455	72%
Black/African American	125	20%
Asian	7	1%
Native American	7	1%
Multi-racial	38	6%
Other	4	1%
Hispanic or Latino/Latina		
No	568	89%
Yes	68	11%
Insurance type		
Has private insurance	348	55%

* Based on non-missing data. The first 52 patients enrolled were not surveyed on their sex due to a coding error in the electronic survey.

**Table 2:** Class means and percentages of covariates.

	Low barriers (60%)	Working barriers (13%)	Financial barriers (10%)	Appointment concerns (8%)	Illness concerns (6%)	Many barriers (2%)
% Female	50%	57%	57%	56%	57%	26%
χ^2^ (1) = 5.82, p = 0.32						
Mean age (1 =18–26; 2 = 27–65; 3 = 65+)	2.13	1.73	1.89	1.91	1.79	1.39
χ^2^ (1) = 65.65, p < 0.001						
% White	66%	74%	66%	47%	47%	31%
χ^2^ (1) = 14.09, p = 0.015						
% Hispanic/Latino(a)	8%	9%	16%	10%	24%	46%
χ^2^ (1) = 9.32, p = 0.097						
% with private insurance	58%	67%	58%	30%	35%	23%
χ^2^ (1) = 22.99, p < 0.001						
% with a primary care provider	92%	84%	82%	81%	85%	85%
χ^2^ (1) = 7.94, p = 0.16						

Note: χ^2^ values represent overall class comparisons.
